# Study on Process Parameters and Lap Ratio for Laser Cladding IN718 Repair of EA4T Steel

**DOI:** 10.3390/ma18214992

**Published:** 2025-10-31

**Authors:** Shaoping Hu, Yanchong Gao, Longfeng Sun, Chao Zhang, Tianbiao Yu

**Affiliations:** 1 School of Mechanical Engineering and Automation, Northeastern University, Shenyang 110819, China; 2190007@stu.neu.edu.cn (S.H.); 18340355586@163.com (Y.G.); zhangchao1@mail.neu.edu.cn (C.Z.); 2 Jiangnan Shipyard (Group) Co., Ltd., Shanghai 201913, China; 18945068082@163.com

**Keywords:** laser cladding, orthogonal experiments, IN718, EA4T steel

## Abstract

Laser cladding offers distinct advantages over traditional manufacturing methods, including low heat input, minimal dilution ratio, dense clad layers, and robust bonding. It is widely employed for surface strengthening of metals to enhance performance and repair failed components, thereby reducing material waste. This study investigates laser cladding repair of EA4T steel, focusing on examining the effects of laser power, scanning speed, and powder feed rate on melt pool dilution ratio and shape factor during cladding of IN718 material onto EA4T steel substrate. Orthogonal experiments were conducted to investigate the combined effects of different process parameters on dilution rate and shape factor. Optimal process parameters were determined by comprehensively evaluating melt pool cross-sectional morphology and internal defects. Based on this, theoretical lap calculations were performed, and the optimal theoretical lap ratio was obtained through experiments. Experiments indicated that the influence of process parameter variations on molten pool morphology parameters is not linear; the combined effects of all factors must be comprehensively considered.

## 1. Introduction

Laser cladding technology involves melting cladding material using a high-energy laser beam to deposit it onto the surface of the substrate, forming a strong metallurgical bond with the base material. This process achieves the objectives of strengthening coatings or performing repairs. This technology is widely applied in the repair and manufacturing of critical components, particularly within the aerospace sector [1,2,3].

Cladding materials exhibit diversity, such as iron-based powders, nickel-based alloy powders, and mixtures of various materials. The cladding powder is uniformly fed into the pipeline through the rotation of the powder feed tray. Under pressure and gas flow, the powder is blown by the powder feeder to the end of the powder feed pipeline [4,5]. To ensure uniform powder feeding, the powder nozzle is typically configured with three or four feed lines, with the convergence point of these lines serving as the laser focal area. The laser, generated by a laser source, is transmitted via optical fiber to the cladding zone. The laser beam irradiates the cladding material and substrate, melting them to form a molten pool [6,7,8]. To maintain real-time synchronization between the powder feed focal point and the laser focal point, both the powder nozzle and the laser are mounted on the same robotic arm. The robotic arm is usually controlled by a robot or machine tool. By programming control sequences, the movement trajectory and speed of the robotic arm are precisely regulated, achieving high-precision control to complete accurate laser cladding forming [9,10]. As the laser beam and powder feeder nozzle move synchronously, the molten pool continuously forms. Upon the laser beam’s withdrawal, the molten pool gradually solidifies, ultimately forming a continuous clad layer. During laser cladding, the control program can be modified based on the characteristics of different cladding materials to alter key process parameters such as laser power, powder feed rate, and scanning speed. This enables precise control over the clad layer’s formation characteristics, reducing defects while further enhancing the clad layer’s properties [11]. For specialized applications demanding exceptional surface properties—such as wear resistance [12] or corrosion resistance [13]—additives can be incorporated into single materials or alloys to achieve superior performance beyond their inherent capabilities. Common additives include rare earth elements and ceramic materials, such as cerium, lanthanum, WC [14], and TiC [15]. Incorporating these elements significantly refines grain size or enhances coating properties through the formation of secondary phases. Laser cladding’s extremely high energy density enables precise control over coating thickness, width, and depth, meeting diverse repair and strengthening requirements. This high energy density results in a smaller heat-affected zone compared to traditional manufacturing methods, generating lower thermal stresses and preventing crack formation or failure caused by excessive thermal stress [16]. 

Laser cladding offers greater flexibility than conventional manufacturing. For applications requiring surface material properties superior to the base material, laser cladding can economically deposit high-performance surface layers onto components to enhance surface characteristics. For severely worn components, laser cladding technology reduces energy consumption by applying high-performance materials, enabling the reuse of components that would otherwise be scrapped. This significantly minimizes material waste and exhibits environmentally friendly characteristics. Given the continuous depletion of Earth’s resources, reducing resource waste holds significant importance [17,18]. G Kim et al. [19] investigated the influence mechanisms on the microstructure and mechanical properties of WC-12Co composite coatings during laser cladding. Results indicate that to enhance the hardness of the clad layer in WC-Co composite coatings, Fe dissolution from the substrate should be minimized to prevent dilution of the clad layer and inhibit the formation of Fe-Co alloy phases. L Tuz [20] investigated the feasibility of producing cobalt-rich plasma transfer arc (PTA) overlay coatings on duplex stainless steel, combining the unique properties of duplex steel with the wear resistance of the coating. The study confirmed the risk of σ-phase formation during duplex steel overlay welding and that elevated temperatures in the carbide layer could induce transition phase formation. Additionally, L Tuz et al. [21] examined the effects of heat input during machining on microstructural transformations and cracking in high-carbon rail steels. Results indicated detrimental microstructural changes within the steel, characterized by the formation of pearlite and transformed ledeburite. H P Boschetti et al. [22] conducted a double-disc test between two rail sections (fully pearlitic and spheroidized microstructure) to investigate the influence of flash-butt welded rail heat affected zone (HAZ) microstructure. Spheroidized cementite microstructure was obtained by heat treatment simulating the softer HAZ region. Results indicated that the spheroidized cementite disc (lower hardness) exhibited a more pronounced surface hardening effect due to deformation. F Pape et al. [23] described a process route for producing axial bearing washers using custom forming technology. Bearing washers were selected for mounting axial roller bearings (type 81212). The manufacturing process began with laser cladding a hard surface made of martensitic chromium-silicon steel (1.4718) onto a substrate of S235 (1.0038) steel. During the cladding process, complex temperature fields and localized thermal gradients inevitably induce residual stresses within the specimen. These stresses significantly influence the microstructural evolution and service properties of the cladding layer [24,25]. It is also noteworthy that the cladding and substrate are typically different alloys. This may lead to defects such as interfacial cracks, pores, or element dilution, thereby degrading the overall performance of the cladding layer [26,27,28].

In summary, the purpose of the research was to investigate the effects of laser power, scanning speed, and powder feed rate on penetration depth, width, and height by laser cladding IN718 material onto an EA4T steel substrate. To achieve a clad layer with morphology closest to the ideal profile, orthogonal experiments were conducted to investigate changes in clad layer morphology. The grey correlation method was employed to select optimal process parameters. The ideal overlap ratio was calculated for the clad layer morphology under these parameters, with experimental results indicating an ideal overlap ratio of 33.6%.

## 2. Test Material Selection and Experimental Design

### 2.1. Matrix and Powder Composition and Parameters

EA4T is a low-alloy structural steel specifically designed for railway vehicle axles. It is characterized by its combination of high strength and high toughness, meeting the stringent safety and reliability requirements demanded during railway vehicle operation. EA4T steel’s excellent mechanical properties, high corrosion resistance, and acceptable impact strength makes it the optimal choice for manufacturing high-speed railway axles [29,30,31]. The chemical composition of EA4T is detailed in Table 1 [32].

Inconel 718 powder is a commonly used nickel-based high-temperature alloy powder, particularly suitable for additive manufacturing due to its weldability [33,34] and high strength at moderate temperatures [35,36]. The high chromium (Cr) and molybdenum (Mo) content in Inconel 718 alloy enables excellent performance in both oxidizing and reducing environments. It demonstrates superior corrosion resistance compared to other nickel-based alloys (such as Inconel 625), particularly in acidic and high-temperature corrosion conditions. Inconel 718 powder forms strong metallurgical bonds with various substrate materials, achieving a low dilution rate and superior cladding quality. Its diffusivity enables strong bonding with the substrate, minimizing defects like cracks and porosity to ensure a dense, high-quality clad layer. Inconel 718 possesses high fatigue strength and resistance to stress corrosion cracking, making it suitable for applications involving repeated stress and vibration, such as aircraft engine blades and turbine discs. These properties render Inconel 718 ideal for extremely demanding environments, delivering outstanding long-term performance. The chemical composition of Inconel 718 is detailed in Table 2.

The Inconel 718 powder used in this experiment has a particle size of 100–270 mesh, within the equipment’s permissible range of 50–300 mesh. Before the experiment, the powder was placed in a drying oven to ensure sufficient dryness and prevent clogging in the powder feed lines. The drying oven was set to 120 °C for a duration of 2 h.

### 2.2. Laser Cladding Forming System

**Figure 1:**
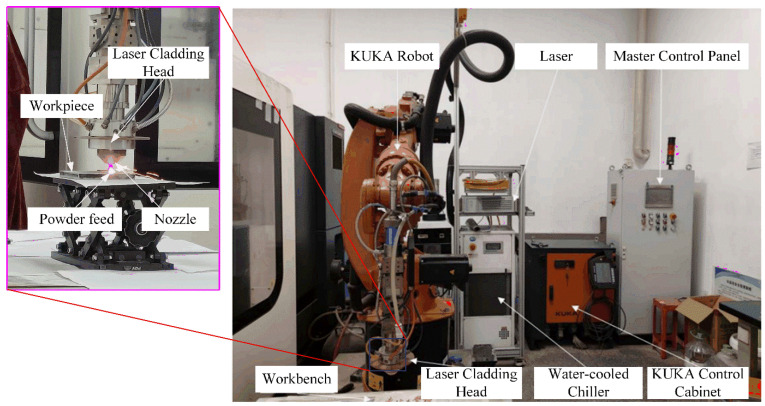
Laser Cladding Experimental Platform.

The experimental setup is illustrated in Figure 1. It comprises a six-degree-of-freedom KUKA robot (KUKA Robotics, Augsburg, Germany), an electrical control cabinet (Zhongke Raycham Laser Tech, Nanjing, China), a fiber laser (IPG Photonics, Oxford, MA, America), a powder feeder (Zhongke Raycham Laser Tech, Nanjing, China), water-cooling equipment(Zhongke Raycham Laser Tech, Nanjing, China), and protective devices (Zhongke Raycham Laser Tech, Nanjing, China) that work in coordination to complete the laser cladding process. The laser beam center emitted by the laser and the powder delivery center from the powder feeder are aligned at the same point in three-dimensional space. The terminal sections of the powder delivery line and the shielding gas line are fixed to the end effector of the KUKA robot. 

(1) KUKA Robot: The KUKA robotic equipment used in the experiment primarily consists of two components: the robotic arm section and the control cabinet section. During operation, the robotic arm is controlled by manipulating the teach pendant on the control cabinet. The main component models and parameters are listed in Table 3.

(2) Fiber Laser: The laser employed in the laser cladding experimental platform is a continuous-wave fiber laser manufactured by IPG Photonics. The model designation is YLR-500-WC, and it delivers an output power of 500 W. Specifically designed for material processing applications, it is widely utilized in industrial manufacturing sectors such as cutting, welding, laser cladding, and heat treatment. Its fiber transmission offers greater flexibility compared to other methods and better accommodates the motion patterns of robotic arms. It provides stable output with minimal energy loss during transmission. Its key parameters and performance characteristics are detailed in Table 4.

(3) Powder Feeding System: The coaxial powder delivery method was employed in this experiment. It delivers powder concentrically around the laser beam. The powder is ejected through a ring-shaped nozzle with 360° uniformly distributed orifices, converging with the laser beam along the central axis. The experimental setup utilizes an RC-PGF-S single-hopper powder feeder. In this system, powder is stored in the hopper and delivered by a rotary disc at its base. It is then transported through the feed line to four nozzles uniformly distributed around the cladding head, where it is deposited onto the substrate to complete the laser cladding process. The RC-PGF-S feeder ensures high precision in powder delivery, which meets diverse process requirements for feed rates and prevents defects caused by feeding instability. The key technical parameters of the RC-PGF-S powder feeder are shown in Table 5.

### 2.3. Laser Cladding Quality Evaluation Standards

After conducting single-pass experiments, the specimens were evaluated to identify the process parameters that best approach the ideal conditions, to obtain a cladding layer characterized by a favorable microstructure and mechanical properties. Following an initial visual inspection of the surface quality, cross-sectional samples were prepared using wire cutting, grinding, and polishing. These cross-sections were then examined under a microscope to measure the clad height, penetration depth, and width. Based on these dimensions, the width-to-height ratio and dilution rate were calculated, enabling a systematic evaluation of the cladding layer quality and supporting subsequent optimization of the process parameters.

**Figure 2:**
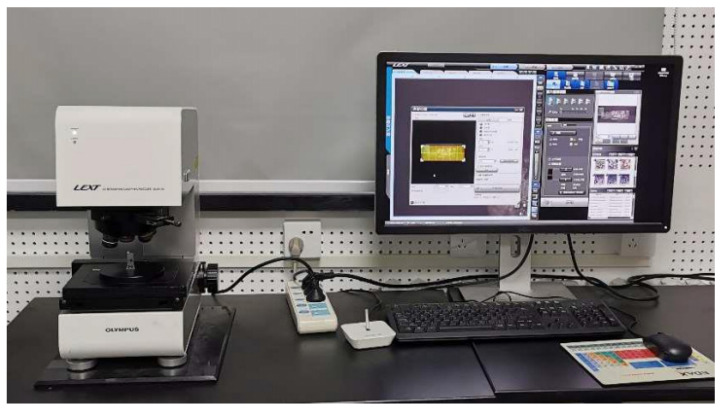
OLS4100 3D Laser Scanning Electron Microscope.

For cross-sectional analysis of the single-pass clad layers, an OLS4100 3D laser scanning confocal microscope (Olympus Corporation, Tokyo, Japan) was employed, as shown in Figure 2. The OLS4100 is a high-precision instrument designed for capturing surface topography and measuring melt pool characteristics. It provides high-resolution three-dimensional imaging, accurate measurement, and non-destructive surface analysis. With its high-quality objectives and excellent repeatability and reliability, the microscope is well-suited for observing and quantifying cladding layer cross-sections.

**Figure 3:**
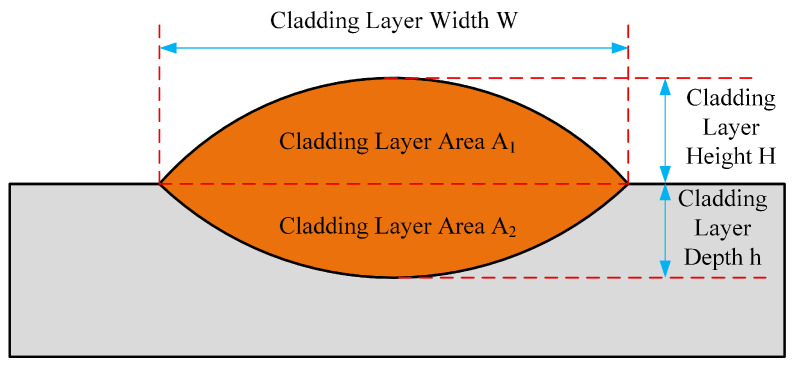
Schematic Diagram of Single-Track Cladding Layer.

The cross-sectional schematic of the clad layer is shown in Figure 3. The geometric dimensions of the clad layer, including clad height, clad depth, and clad width, are denoted by the letters H, h, and W, respectively, in this paper.

In the laser cladding, the shape factor ξ serves as a key evaluation metric for the melt pool cross-section, reflecting the ratio between the clad layer width W and the clad layer height H. In this paper, the shape factor ξ is defined as the ratio of the cladding layer width W to the clad layer height H. The formula for calculating the shape factor ξ is:

(1) $$\xi \;=\;\frac{W}{H}$$

There is no single definitive value for the optimal shape factor in laser cladding; its “best value” varies depending on the application scenario. Generally, the optimal shape factor is typically maintained within the range of 3–5. This ensures an optimal cross-sectional shape while guaranteeing uniformity and density of the clad layer, thereby minimizing defects. 

During the laser cladding process, metallurgical bonding requires a certain degree of material exchange between the cladding layer and substrate. The dilution rate η serves as a critical metric for assessing the incorporation of substrate material into the clad layer during laser cladding, directly influencing the bonding properties of the clad layer. The dilution rate η is typically defined as the volume fraction of substrate material within the clad layer, expressed as the ratio of the clad layer area A_2_ to the total clad layer area A_1_ + A_2_. This can be represented by the formula:

(2) $$\eta \;=\;\frac{A_{2}}{A_{1}+A_{2}}$$

However, in practical operations, accurately calculating the areas of coating layers A_1_ and A_2_ is challenging due to their irregular shapes. Therefore, this paper employs a simplified formula to calculate the dilution rate. Since the dilution rate is an area ratio, clad depth h is substituted for clad layer area A_2_, and clad height H is substituted for clad layer area A_1_ in the calculation. The simplified dilution rate formula is as follows:

(3) $$\eta =\frac{h}{H+h}$$

The dilution rate exerts varying effects on the cladding layer. At low dilution rates, less substrate material enters the cladding layer, minimizing its influence on the cladding layer properties. However, excessively low dilution rates may compromise the metallurgical bonding between the cladding layer and substrate, increasing the risk of cladding layer detachment. At higher dilution rates, the base material significantly affects the cladding layer material, diluting it to a greater extent. This situation may consequently compromise the superior properties of the cladding material [37].

### 2.4. IN718 Laser Cladding on EA4T Steel: Single-Factor Experimental Design

In laser cladding experiments, numerous factors influence outcomes, including laser power, scanning speed, powder feed rate, defocus amount, substrate preheating temperature, and shielding gas flow rate. Given the multitude of parameters and considerations for experimental efficiency and significant parameter effects, this study focuses solely on the impact of laser power P, scanning speed V_s_, and powder feed rate V_f_ on the cladding layer. The laser used in this experiment has a spot diameter of 1 mm and a maximum power of 500 W. Therefore, the maximum laser power in single-factor experiments should not exceed this value. Additionally, the shielding gas flow rate is set to 15.0 L/min, the powder feed gas flow rate to 7.0 L/min, and the substrate temperature to room temperature. Based on these conditions, single-factor experiments are designed. The single-factor experimental design is shown in Table 6.

## 3. Analysis of Single-Factor Experiment Results

### 3.1. Laser Power Single-Factor Experiment

**Figure 4:**
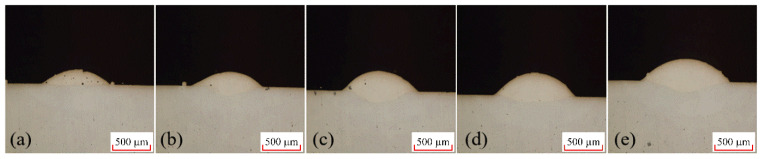
Morphology of Clad Layer at Different Laser Powers, (**a**) 300 W; (**b**) 340 W; (**c**) 380 W; (**d**) 420 W; (**e**) 460 W.

In the laser cladding experiments, the laser power P is one of the most critical process parameters, directly affecting the quality of the clad layer. By conducting a single-factor experiment on laser power, we can preliminarily verify whether the selected power range effectively melts both the cladding powder and the substrate material, laying the groundwork for subsequent orthogonal experiments. Based on equipment parameters, a single-factor laser power experiment was conducted with five levels: 300 W, 340 W, 380 W, 420 W, and 460 W. The cross-section of the clad layer from this single-factor laser power experiment is shown in Figure 4. Measurements of the cladding layer interface under different laser powers yielded the fusion height H, fusion depth h, and fusion width W. Further calculations based on the aspect ratio and dilution rate formulas described above produced the molten pool data for each laser power.

**Figure 5:**
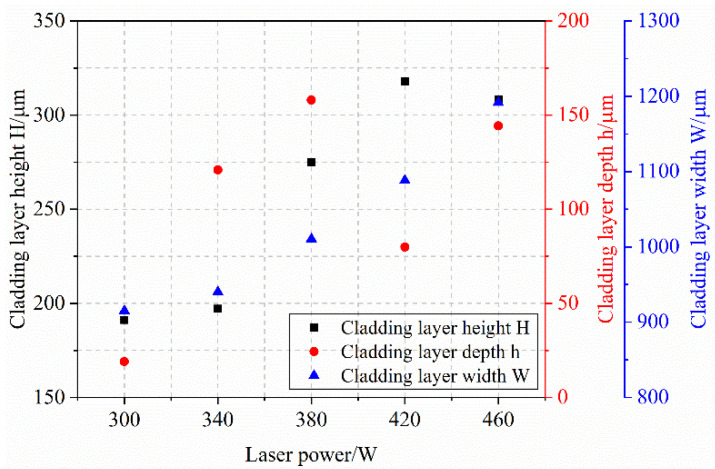
Effect of Laser Power on Melt Height, Melt Depth, and Melt Width.

**Figure 6:**
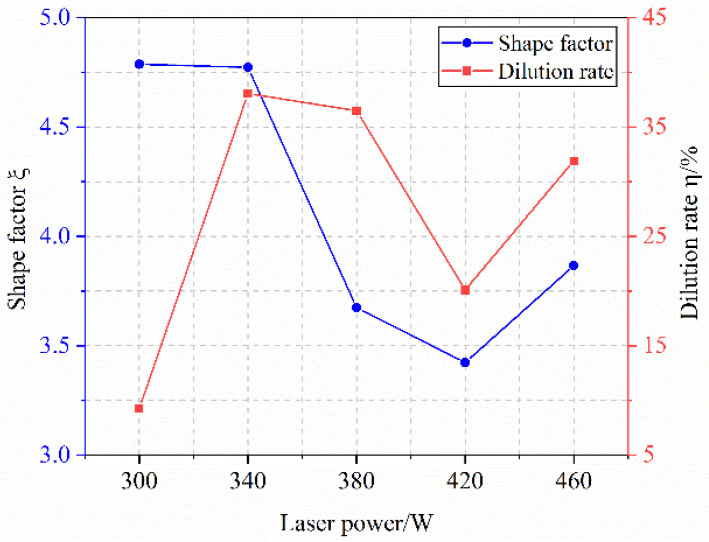
Effect of Laser Power on Shape Factor and Dilution Rate.

Figure 5 depicts the influence of laser power on melt height, depth, and width, while Figure 6 presents its effect on the shape factor and dilution rate. As shown in Figure 5, the melt height increases from 191.15 μm to 317.97 μm with rising laser power, which is attributed to enhanced energy input that melts more cladding powder and elevates the melt pool surface. The penetration depth initially rises from 19.50 μm to 158.00 μm, with a notable surge between 300 W and 380 W, due to greater energy absorption by the melt pool and more substrate melting. However, beyond a certain power level, penetration depth decreases (e.g., at 420 W and 460 W). This reversal is caused by changes in melt pool morphology and increased material fluidity above the pool, which accelerates flow and reduces heat transfer into the substrate—a trend corroborated by the declining shape factor. Meanwhile, melt width consistently expands with laser power, as higher energy input melts more powder, thereby widening the melt zone.

As laser power varies, corresponding changes are observed in both the shape factor and dilution rate. The shape factor initially decreases and then increases with higher laser power. At lower powers (300 W and 400 W), insufficient energy leads to more powder melting than substrate melting, causing the molten powder to adhere to the surface with negligible penetration, as illustrated in Figure 4a. In contrast, at 460 W, elevated surface temperature enhances fluidity, resulting in a larger shape factor. The dilution ratio first increases and then decreases overall with laser power. Due to the coaxial powder feeding method, penetration depth remains nearly constant beyond 340 W (around 130 μm), while the weld height continues to rise. This leads to a stable numerator but a growing denominator in the dilution formula, thereby reducing the dilution ratio.

In addition to geometric parameters, melt pool quality also improves with increasing laser power. Figure 4 shows that the cladding surface becomes smoother, as higher power ensures complete powder melting, thereby reducing agglomeration and incomplete fusion that occur at low power. Porosity, a key indicator of clad integrity, also decreases with rising laser power from 300 W to 380 W. By 420 W, no porosity is detected in cross-sections. This is attributed to adequate energy input, which promotes full melting and degassing, whereas insufficient energy results in incomplete bonding and the trapping of gas.

### 3.2. Single-Factor Experiment on Scanning Speed

**Figure 7:**
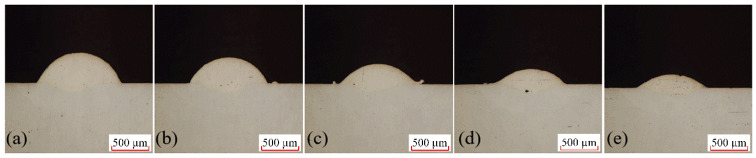
Single-factor experiment on scanning speed and molten pool cross-section, (**a**) 3 mm/s; (**b**) 4 mm/s; (**c**) 5 mm/s; (**d**) 6 mm/s; (**e**) 7 mm/s.

In the laser cladding experiments, scanning speed is a critical process parameter that directly impacts the quality of the clad layer. With all other parameters held constant, distinct scan speeds of 3 mm/s, 4 mm/s, 5 mm/s, 6 mm/s, and 7 mm/s were set. Through experimentation, the single-factor scan speed test cross-section is shown in Figure 7. Measurements of the cladding layer interface under different scanning speeds yielded the fusion height H, fusion depth h, and fusion width W. Further calculations based on the aspect ratio and dilution rate formulas described above produced the molten pool data for each scanning speed.

**Figure 8:**
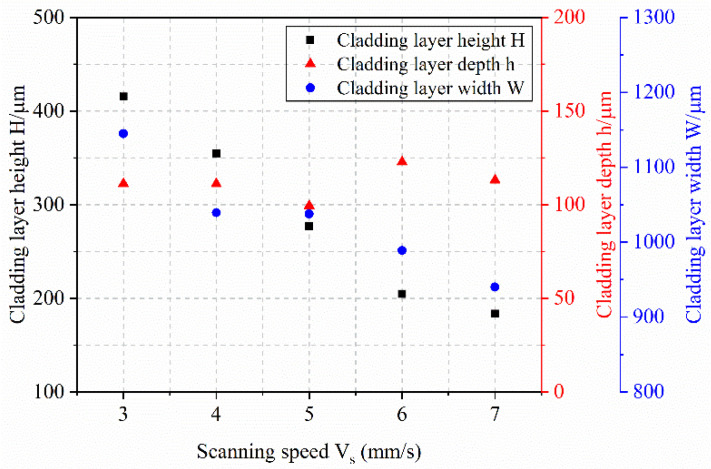
Effect of Scanning Speed on Melt Height, Melt Depth, and Melt Width.

Figure 8 illustrates that as the scanning speed increases from 3 mm/s to 7 mm/s, the melt height gradually decreases. This trend occurs because a higher scanning speed spreads the same powder mass over a longer track length per unit time, thereby reducing the powder mass per unit length and resulting in a lower melt height. Although the penetration depth fluctuates with increasing scanning speed, it remains consistently around 110 μm, indicating a relatively minor influence of scanning speed on penetration depth under the experimental conditions. Meanwhile, the melt width also decreases gradually with increasing scanning speed, which is attributed to the reduced energy input per unit area.

**Figure 9:**
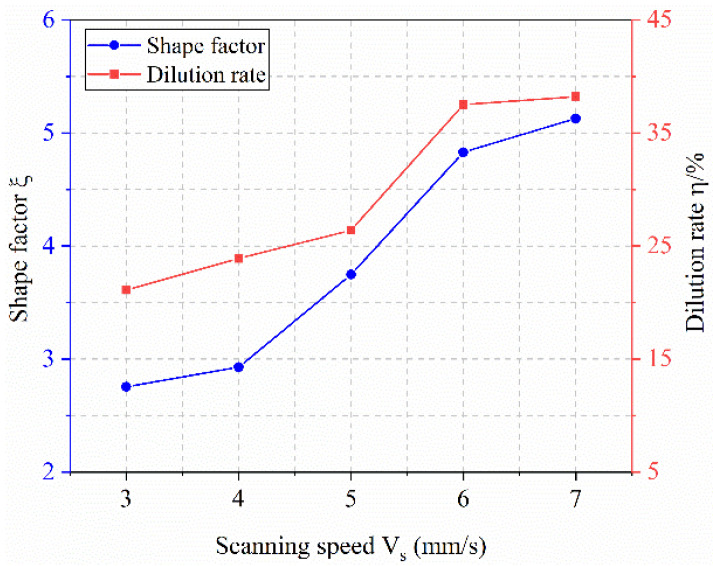
Effect of Scanning Speed on Shape Factor and Dilution Rate.

As shown in Figure 9, the shape factor increases gradually with scanning speed. This trend arises because the scanning speed influences melt height more significantly than melt width. Specifically, the melt height decreases markedly with increasing speed, while the melt width exhibits a comparatively smaller change. As a result, the numerator (melt height) in the shape factor expression decreases less than the denominator (melt width), leading to an overall increase in the shape factor. The dilution rate also rises with increasing scanning speed. Although the penetration depth remains relatively stable under higher speeds, the melt height decreases considerably. This reduction in melt height causes the denominator in the dilution rate formula to decrease, thereby increasing the dilution rate.

### 3.3. Single-Factor Experiment on Powder Feed Rate

**Figure 10:**
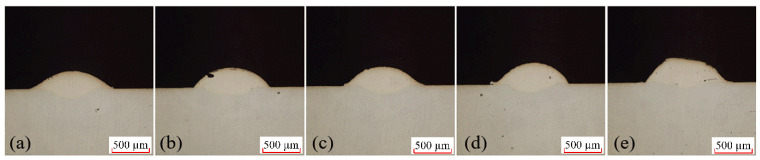
Single-factor Experiment on Powder Feed Rate and Melt Pool Cross-section, (**a**) 0.8 r/min; (**b**) 0.9 r/min; (**c**) 1.0 r/min; (**d**) 1.1 r/min; (**e**) 1.2 r/min.

The powder feed rate is essential to investigate the effect of powder feed rate on weld pool quality. Single-factor experiments were conducted by setting the powder feed rate to 0.8 r/min, 0.9 r/min, 1.0 r/min, 1.1 r/min, and 1.2 r/min, respectively, while keeping all other parameters constant. The cross-sections of the cladding layers obtained are shown in Figure 10. Measurements of the cladding layer interface under different powder feed rates yielded the fusion height H, fusion depth h, and fusion width W. Further calculations based on the aspect ratio and dilution rate formulas described above produced the molten pool data for each powder feed rate.

**Figure 11:**
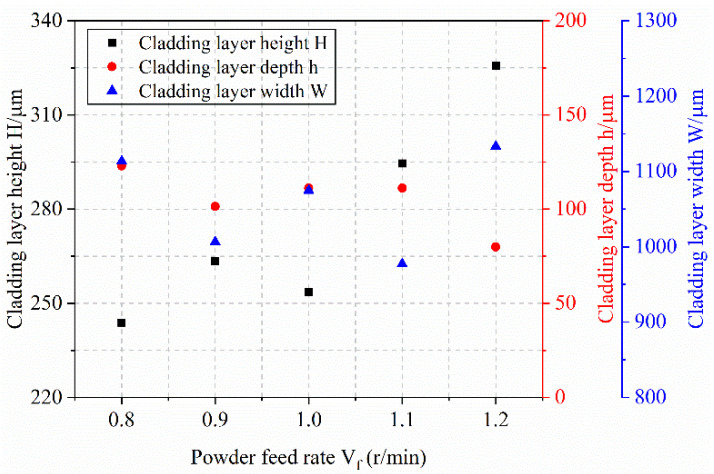
Effect of Powder Feed Rate on Melt Height, Melt Depth, and Melt Width.

Figure 11 illustrates the influence of powder feed rate on melt dimensions. As the feed rate increases from 0.8 r/min to 1.2 r/min, the melt height rises progressively due to the greater amount of powder supplied per unit time. In contrast, the penetration depth decreases, as more laser energy is absorbed by the increased powder volume, reducing the energy available for substrate melting. Meanwhile, the melt width fluctuates slightly but generally stabilizes around 1040 μm, indicating a limited effect of feed rate on this parameter.

**Figure 12:**
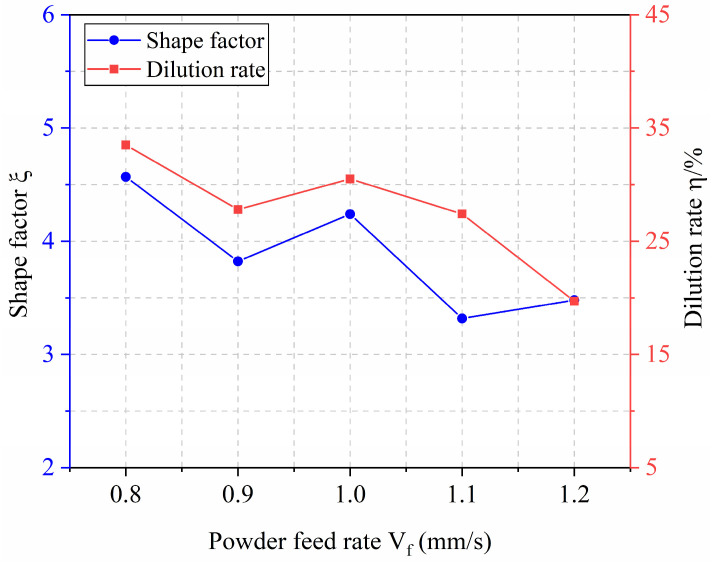
Effect of Powder Feed Rate on Shape Factor and Dilution Rate.

Figure 12 shows the corresponding variations in shape factor and dilution rate. The shape factor generally decreases with increasing powder feed rate. This trend occurs because the clad width remains stable while the clad height increases, leading to a nearly constant numerator but a growing denominator in the shape factor expression. Similarly, the dilution rate decreases as the powder feed rate rises. This is attributed to the combined effect of a decreasing penetration depth (numerator) and an increasing clad height (denominator) in the dilution formula.

## 4. Analysis of Orthogonal Experiment Results

### 4.1. Orthogonal Experimental Design

**Figure 13:**
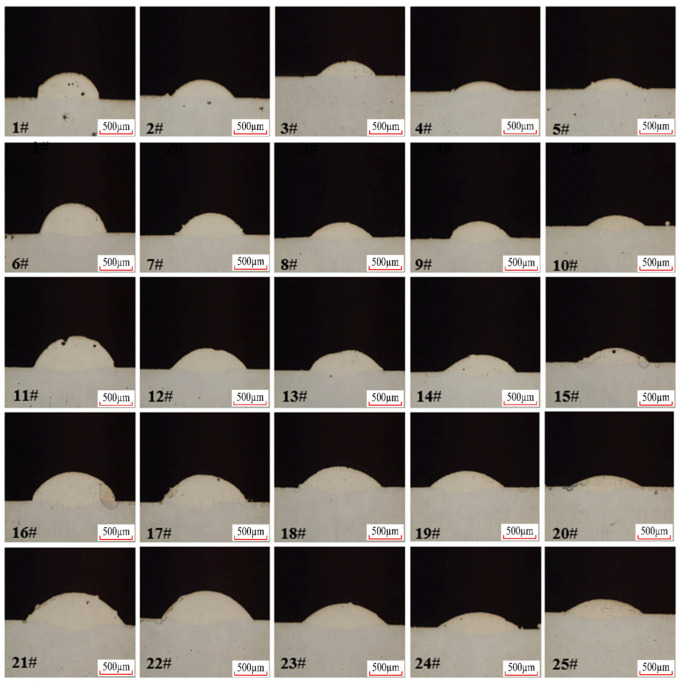
Cross-sectional morphology of single-track cladding layer from orthogonal experiment.

The three factors most significantly influencing laser cladding quality are laser power, scanning speed, and powder feed rate. An orthogonal experimental design was conducted for these three factors, with five levels set for each factor. Test specimens were processed to obtain their cross-sectional morphology. Based on the cross-sectional results, the melt height, penetration depth, and width of the melt pool were measured. Further calculations yielded the shape factor and dilution rate. The orthogonal experimental design and measured data are shown in Table 7, while the cross-sectional morphology is depicted in Figure 13.

Based on previous research, in terms of microstructure, both the matrix and coating are primarily composed of FCC phases. The matrix exhibits an equiaxed grain structure with minor twinning. Additionally, the coating microstructure features dendritic structures and spherical precipitates. The presence of δ phase inhibits grain growth in the coating, resulting in grain sizes smaller than those in the matrix. Compared to the EA4T steel substrate, the mechanical and tribological properties of the Inconel 718 coating are significantly enhanced. This improvement stems primarily from the following mechanisms: First, the rapid solidification process during laser cladding enables elements such as Cr, Mo, and Nb from the Inconel 718 powder to dissolve into the EA4T steel substrate, generating a pronounced solid solution strengthening effect; Second, under thermal cycling, the coating precipitates a large amount of nanoscale strengthening phases. These phases enhance material properties by impeding dislocation movement, providing excellent dispersion strengthening and grain refinement effects. The synergistic action of these factors ensures good metallurgical bonding between the coating and substrate, endowing the coating with higher load-bearing capacity and deformation resistance. Furthermore, the abundant Cr, Mo, and other elements in the coating readily form stable, dense oxide films under frictional heat. This layer not only mitigates adhesive wear but also provides solid lubrication to reduce the coefficient of friction, thereby offering comprehensive protection to the substrate. Consequently, the Cr and Mo elements in the coating also contribute to enhancing the surface wear resistance and corrosion resistance of the coating [38].

### 4.2. Calculation of Range for Experimental Results

Range analysis is a commonly used method in orthogonal experiments for assessing the influence of various factors on experimental outcomes. This approach involves calculating the average response value for each factor level, with the range defined as the difference between the highest and lowest of these averages. A larger range indicates a stronger influence of the factor on the result, while a smaller range suggests a weaker effect. By comparing range values, this method allows for the preliminary identification of statistically significant factors. Calculations performed on the experimental data in Table 7 yielded the ranges shown in Table 8.

Among these, K_Hi_, K_hi_, K_Wi_, K_ξi_, and K_ηi_ represent the average values at a given level under different factors, while R_H_, R_h_, R_W_, R_ξ_, and R_η_ denote the respective ranges of variation in weld height H caused by laser power P, scanning speed V_S_, and powder feed rate V_f_ under a single factor. As shown in Table 8, the order of influence of each process parameter on the geometric characteristics of the molten pool is as follows: Pool height is most significantly affected by scanning speed, followed by laser power and powder feed rate; penetration depth is primarily controlled by laser power, with powder feed rate and scanning speed having secondary effects; pool width is also dominated by laser power, with scanning speed and powder feed rate exerting weaker influences; shape factor is most significantly affected by scanning speed, followed by powder feed rate, with laser power having the least impact. Dilution rate is successively influenced by laser power, scanning speed, and powder feed rate. Overall, under the experimental conditions, the ranking of parameter influence on the molten pool is: laser power > scanning speed > powder feed rate.

### 4.3. Range Analysis

**Figure 14:**
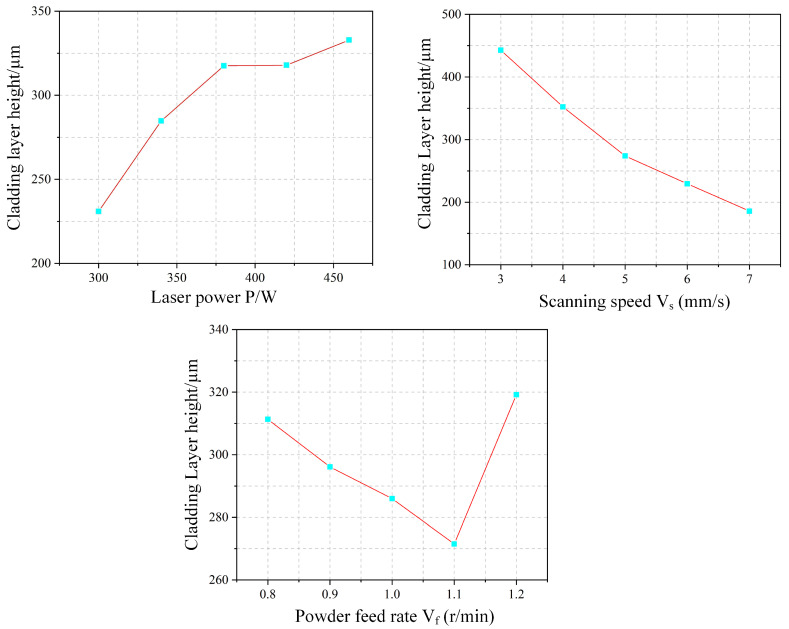
Experimental Effect Diagram of Cladding Layer Height.

Figure 14 illustrates the variation in cladding height (H) with process parameters. The cladding height increases with laser power, as higher power melts more powder, thereby elevating the deposit. However, under a constant powder feed rate, the available powder is limited, leading to a diminishing rate of height increase at higher power levels. In contrast, the melt height decreases with increasing scan speed. Although the powder feed rate per unit time remains unchanged, a higher speed spreads the powder over a larger area, reducing the powder density per unit length and consequently the melt height. This relationship exhibits a stable, near-linear inverse proportionality. Unexpectedly, the melt height shows an overall decrease with increasing powder feed rate. This deviation from the expected trend is attributed to insufficient laser energy to fully melt the excess powder at higher feed rates. As a result, unmelted particles are either ejected from the melt pool or carried away by the shielding gas, reducing the effective deposition height.

**Figure 15:**
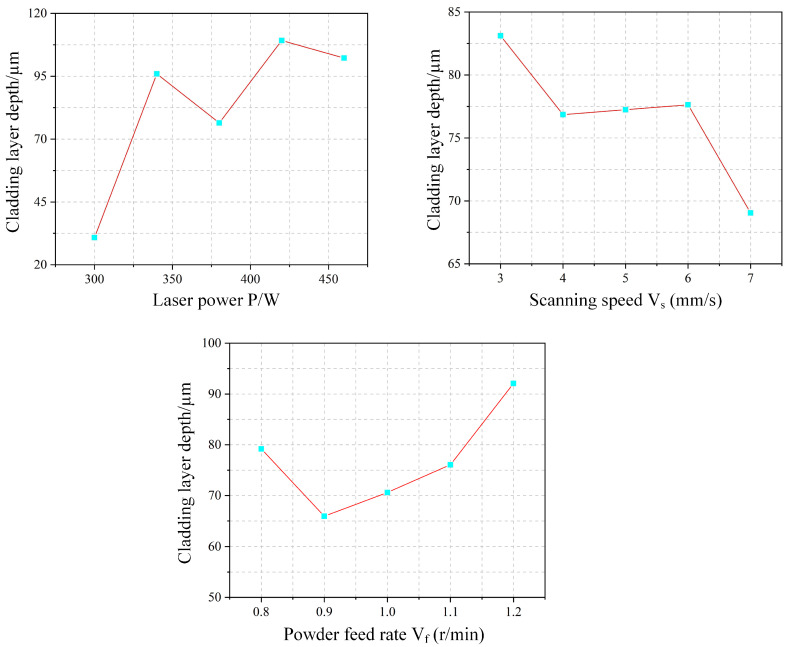
Experimental Effect Diagram of Cladding Layer Depth.

The experimental effect curve for cladding depth h is shown in Figure 15. Overall, the cladding depth (h) exhibits a gradual increase with rising laser power. Higher laser power delivers more energy to the molten pool. While part of this energy melts additional powder—increasing pool height and width—another portion penetrates deeper into the substrate, resulting in greater penetration depth. In contrast, penetration depth generally decreases with increasing scanning speed, which reduces the energy input per unit area and thus limits substrate melting. The relationship between powder feed rate and penetration depth is more complex, showing an initial decrease followed by an increase. At lower feed rates, more ejected powder partially shields the substrate from direct laser irradiation, reducing the penetration depth. As the feed rate further increases, however, a larger volume of melted powder contributes to the molten pool. During subsequent cooling, heat is transferred downward into the substrate, promoting additional melting and consequently increasing the penetration depth.

**Figure 16:**
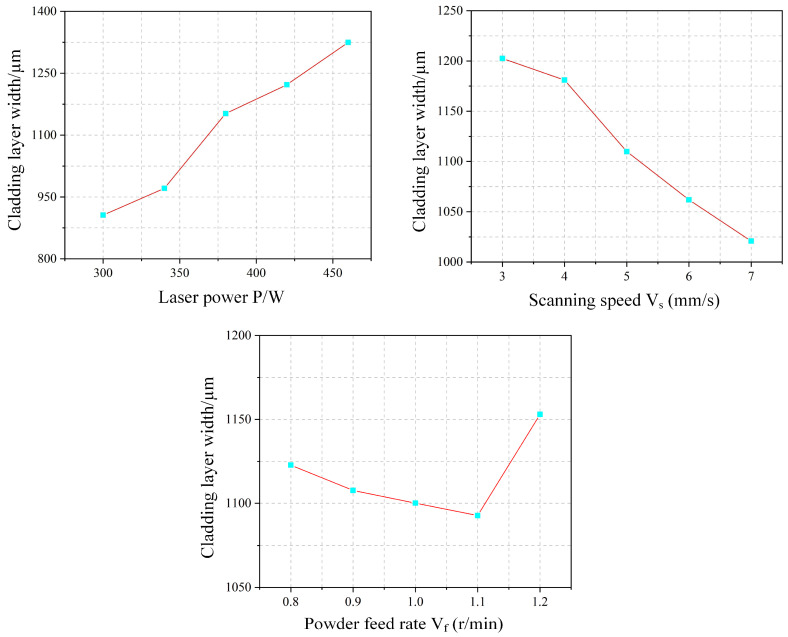
Experimental Effect Diagram of Cladding Layer Width.

Figure 16 illustrates the experimental results for the clad layer width (W). The cladding width increases gradually with laser power, as higher power provides more energy to the melt pool. This enhances powder melting and enlarges the pool’s cross-sectional area. Moreover, improved metal fluidity due to complete melting promotes outward spreading, further widening the clad layer. In contrast, the melt width decreases with increasing scanning speed. This trend is attributed to the reduction in energy input per unit area, which can lead to incomplete powder melting and accumulation over the pool, constraining width expansion. Additionally, the decreased powder mass per unit area at higher speeds contributes to a narrower deposition profile. The relationship between powder feed rate and melt width is non-monotonic, showing an initial decrease followed by an increase. At lower feed rates, insufficient melting causes partially melted powder to accumulate above the pool, reducing effective width. However, at a feed rate of 1.2 r/min, the melt width increases abruptly, likely due to excessive powder accumulation deteriorating cladding quality, where inadequate laser melting leads to irregular powder stacking and apparent width expansion.

**Figure 17:**
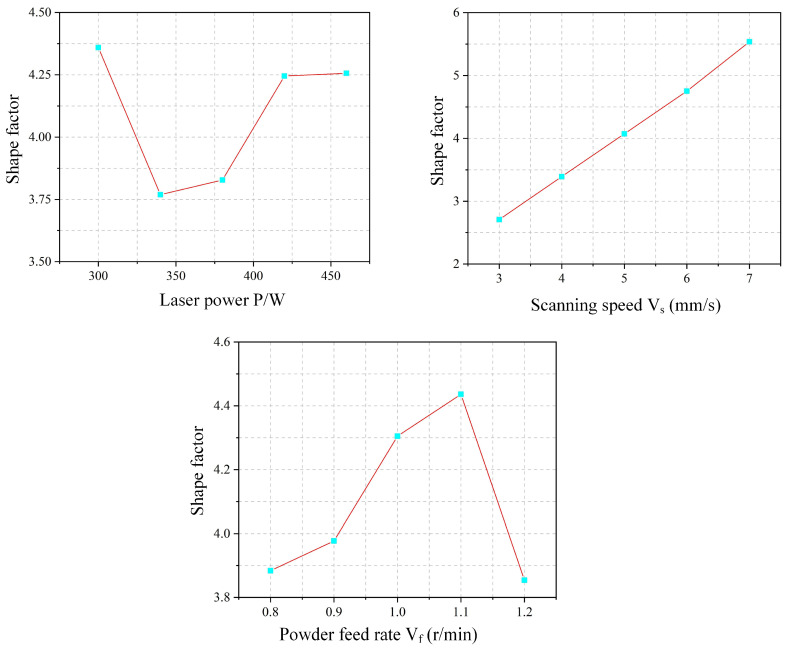
Shape Factor Experimental Effect Diagram.

The experimental effect curve of the shape factor ξ is shown in Figure 17. As laser power increases, the shape factor initially decreases and then increases. At lower power levels, incomplete powder melting leads to accumulation above the melt pool, significantly increasing the melt height with little change in width, thereby reducing the shape factor. As power further increases, complete melting results in a flatter pool morphology, raising the shape factor. The shape factor increases linearly with scanning speed. Higher speeds reduce the powder mass per unit area, lowering the clad height, while the melt width remains relatively stable. This consistent height reduction under nearly constant width leads to the linear rise in the shape factor. With increasing powder feed rate, the shape factor first increases and then drops sharply. At moderate feed rates, sufficient energy enables complete melting, widening the pool and raising the shape factor. However, at 1.2 r/min, inadequate laser energy leads to incomplete melting and powder accumulation above the pool, causing a sharp decrease in the shape factor.

**Figure 18:**
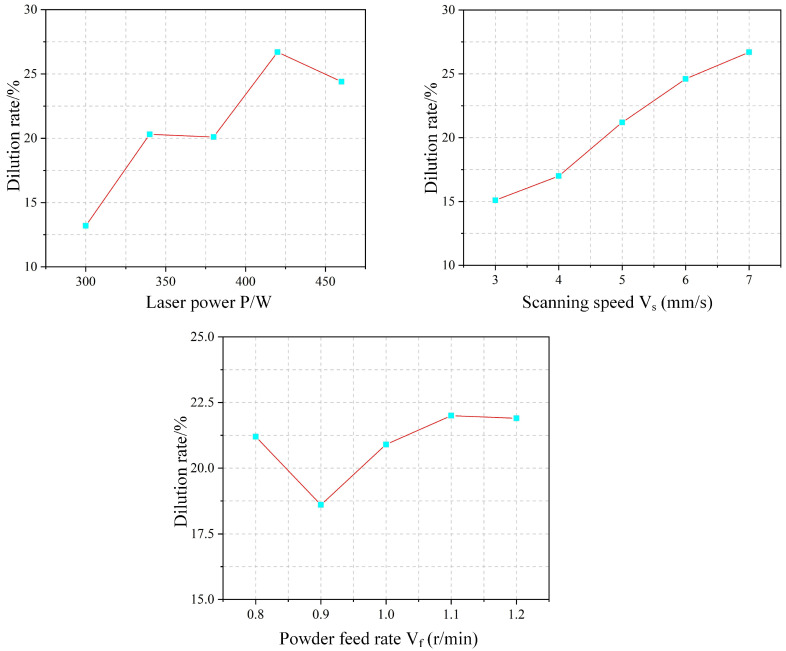
Effect Diagram of Dilution Rate Experiment.

The experimental effect curve of the dilution rate is shown in Figure 18. The dilution rate generally increases with laser power, as higher power introduces more energy into the melt pool, enhancing penetration depth and thus raising dilution. With increasing scan speed, the dilution rate also gradually rises. This is because higher speeds reduce both the powder mass per unit area and the laser energy input, allowing more energy to be directed toward substrate melting, which increases penetration depth and thereby dilution. In contrast, the dilution rate remains largely stable as the powder feed rate increases. Although more powder elevates the clad height, the associated heat dissipation leads to deeper substrate melting. The resulting simultaneous increase in both penetration depth and clad height maintains a nearly constant dilution rate.

## 5. Parameter Optimization and Results Analysis

### 5.1. Optimization Process of the Grey Relational Degree Method

This study demonstrates that both the shape coefficient and dilution rate significantly impact coating quality and require optimization. Signal-to-noise ratio calculation methods vary depending on the response variable and can be categorized into three types: higher values are better, lower values are better, and target value optimization. Regarding the shape coefficient, as analyzed earlier, excessively high or low values impair coating quality and subsequent lap test performance. Therefore, based on actual experimental results, a shape factor of 5 is selected as the optimal target value for optimization. For the dilution rate, both excessively high and low values yield suboptimal experimental outcomes. A dilution rate of 30% was set as the optimization target. Calculation expressions vary according to different requirements. The expressions for “higher is better,” “lower is better,” and “target value optimal” are shown in Equations (4), (5) and (6), respectively:

(4) $$S/N=-10\log_{10}\left(\frac{1}{n}\sum_{i=1}^{n}\frac{1}{{y_{i}}^{2}}\right)$$

(5) $$S/N=-10\log_{10}\left(\frac{1}{n}\sum_{i=1}^{n}{y_{i}}^{2}\right)$$

(6) $$S/N=-10\log_{10}\left(\frac{1}{n}\sum_{i=1}^{n}{\left(y_{i}-y\right)}^{2}\right)$$

Here, *n* denotes the number of experiments, and *y* indicates the optimal value when the target value is optimal. The target values for the shape factor and dilution rate to be optimized are calculated using Equation (6). The *S/N* ratios of the computed shape factor and dilution rate are normalized. Different normalization formulas are applied according to varying requirements: for parameters where larger values are better, Equation (7) is used; for parameters where smaller values are better, Equation (8) is used [39]. Equations (7) and (8) are as follows:

(7) $${X_{i}}^{*}=\frac{S/N_{i}-S/N_{\min}}{S/N_{\max}-S/N_{\min}}$$

(8) $${X_{i}}^{*}=\frac{S/N_{max}-S/N_{i}}{S/N_{\max}-S/N_{\min}}$$

After normalizing the shape factor and dilution rate calculations, the absolute differences must also be computed. Since the values have been normalized, their ideal values are scaled to 1. The calculation formula for the grey correlation coefficient is shown in Equation (9). Here, ζ represents the distribution coefficient, with ζ = 0.5 in the calculation. Δmin denotes the minimum absolute difference among all experimental groups, while Δmax indicates the maximum absolute difference among all experimental groups:

(9) $${\mathrm{GRC}}_{i}\left(k\right)=\frac{\Delta \min\;+\;\zeta \Delta \max}{\Delta_{i}\left(k\right)\;+\;\zeta \Delta \max}$$

The final step in the grey correlation analysis method is to calculate the grey correlation rank. The formula is shown in Equation (10). The grey correlation degree for each experimental group is ultimately obtained and sorted. The calculation results and ranking are presented in Table 9:

(10) $${\mathrm{GRG}}_{i\;}=\;\frac{1}{2}\sum_{i=1}^{n}{\mathrm{GRC}}_{i}\left(k\right)$$

By checking the calculated grey correlation coefficients, it was found that the 25th orthogonal experimental group exhibited the highest grey correlation value. According to prior calculations of the shape coefficient and dilution rate, the 25th orthogonal experimental group yielded a shape coefficient of 5.396 and a dilution rate of 29.8%. These values closely approached the desired shape coefficient of 5 and dilution rate of 0.3. Therefore, subsequent experimental parameters were selected as P = 460 W, V_s_ = 7 mm/s, and V_f_ = 1.1 r/min.

### 5.2. Analysis of the Effect of Combination Strategies on the Cladding Layer Model

In practical applications, multiple passes are performed to form a larger cladding layer for coating or repair purposes. This requires overlapping individual cladding passes to achieve a better cladding surface. The overlap ratio reflects the proportion of lateral overlap between adjacent cladding layers, defined as the ratio of overlap width to single cladding pass width, expressed as a percentage [40,41]. Its calculation formula is shown in Equation (11):

(11) $$\lambda =\frac{W-d}{W}$$

**Figure 19:**
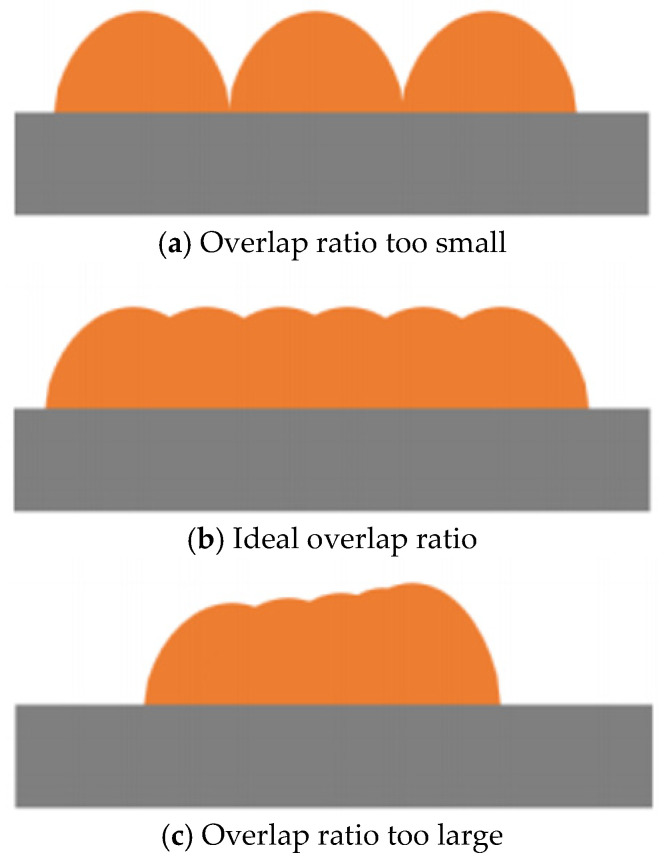
Different types of overlap results.

When performing multi-pass laser cladding, both excessive and insufficient overlap ratios adversely affect the overall performance of the clad layer. Calculating the overlap ratio is necessary to achieve an optimal value that facilitates subsequent experiments or ensures clad layer performance. Figure 19 illustrates three types of overlap results.

**Figure 20:**
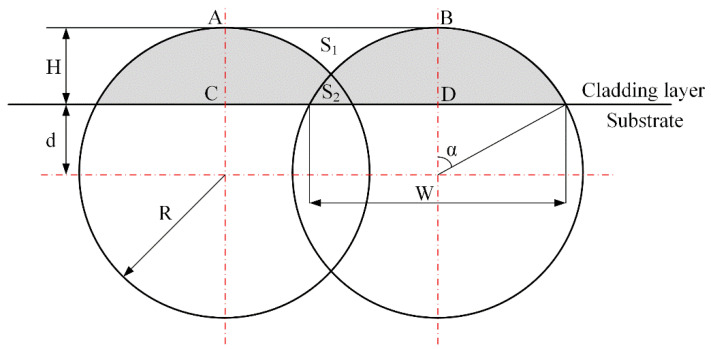
Theoretical Lap Model.

Calculating the overlap ratio involves area computations, and since the shape of the molten pool above the substrate is irregular, the calculation presents significant challenges. Therefore, the upper portion of the molten pool is idealized as a segment of a circle, with the ideal overlap model shown in Figure 20. The principle is that the overlapping area S2 of a single cladding layer during overlap equals the gap area S1 between two single layers, ultimately ensuring a flat upper surface of the overlap. Therefore, it is necessary to calculate the sizes of S1 and S2 to ensure they are equal.

Based on the geometric relationships shown in the diagram and the Pythagorean theorem, we have:

(12) $$R^{2}={\left(\frac{W}{2}\right)}^{2}+{(R-H)}^{2}$$

First, calculate the area of the cladding layer above the molten pool (the area of a single cladding pass). The formulas for angle α, sector area, and triangle area are as follows:

(13) $$\alpha =\arccos\left(\frac{d}{R}\right)$$

(14) $$A_{\mathrm{Sector}}=\frac{2\alpha }{2\pi }\times \pi R^{2}$$

(15) $$A_{\mathrm{triangle}\;\mathrm{area}}=\frac{1}{2}R^{2}\sin(2\alpha )$$

The area of the arc is:

(16) $$A_{\mathrm{arc}}=A_{\mathrm{Sector}\;}-A_{\mathrm{triangle}\;\mathrm{area}}$$

Once again, approved:

(17) $$S_{ABCD}=S_{arc}$$

**Figure 21:**
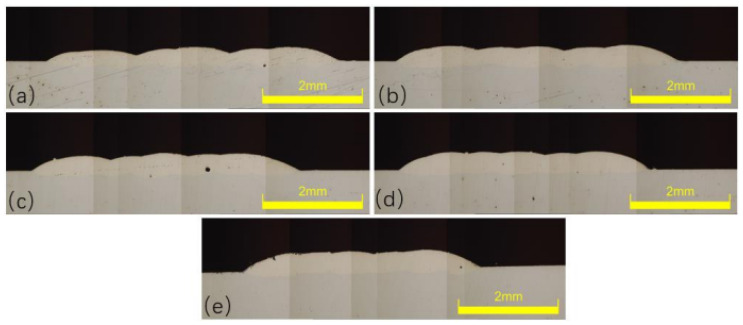
Overlap Ratio Test Image, (**a**) 27.6%; (**b**) 29.6%; (**c**) 31.6%; (**d**) 33.6%; (**e**) 35.6%.

The final dilution rate obtained was approximately 31.6%. Overlap tests were conducted at concentrations of 27.6%, 29.6%, 31.6%, 33.6%, and 35.6%, with 31.6% as the midpoint value and 2% as the interval value. The resulting test images are shown in Figure 21.

The evaluation metric for a single-pass cladding layer is the height-to-width ratio, defined as the ratio of the difference between the maximum and minimum heights of the single-pass cladding layer to its maximum height, as shown in Equation (18):

(18) $$\theta =\frac{H_{\max}-H_{\min}}{H_{\max}}$$

After the calculation, Table 10 is obtained as follows. Based on the aspect ratio, it can be observed that a lap ratio of 33.6% yields better flatness.

## 6. Discussion

This study systematically developed and optimized a laser cladding strategy for depositing Inconel 718 onto EA4T railway axle steel, presenting a novel and sustainable approach for component repair and surface enhancement.

The principal novelty of this work lies in the integrated multi-stage optimization framework. This research employed a synergistic combination of single-factor experiments, orthogonal design, and Grey Relational Analysis (GRA) coupled with Signal-to-Noise ratio analysis to simultaneously optimize two conflicting quality metrics: the shape factor and the dilution rate. The successful identification of an optimal parameter set (P = 460 W, V_s_ = 7 mm/s, V_f_ = 1.1 r/min) that yields a shape factor of 5.396 and a dilution rate of 29.8%—values that closely align with the predefined targets of 5 and 30%, respectively—demonstrates the efficacy of this methodology. This robust data-driven strategy ensures a clad layer with an ideal cross-sectional profile for subsequent overlapping and superior metallurgical bonding, effectively balancing the risk of delamination (from low dilution) with the loss of cladding material properties (from excessive dilution).

Furthermore, the strategic selection of a 33.6% overlap ratio, determined through a systematic evaluation of surface flatness via the aspect ratio, underscores the practical applicability of this research. This approach moves beyond single-track deposition to address the critical challenge of achieving consistent, high-quality multi-track cladding, which is a prerequisite for repairing large-area components like railway axles.

From a sustainability perspective, the proposed laser cladding repair technique offers significant environmental and economic advantages over traditional replacement methods. Firstly, it aligns with the core principles of the circular economy by enabling the repair and reuse of high-value EA4T steel components, thereby drastically reducing material consumption and waste generation associated with manufacturing new axles. The energy-intensive process of mining, refining, and forging new steel is partially circumvented. Secondly, the process itself is optimized for resource efficiency. The precise control over parameters like laser power and powder feed rate minimizes material waste (unmelted powder can often be recycled) and reduces energy consumption by avoiding excessive power inputs that do not contribute to quality. By extending the service life of critical railway components, this repair strategy reduces the lifecycle carbon footprint and costs, contributing to more sustainable railway maintenance operations. Future work will involve a quantitative lifecycle assessment to quantify further these environmental benefits and the validation of the repaired axles under full-scale mechanical testing to solidify the technology’s readiness for industrial adoption.

## 7. Conclusions

This study focuses on analyzing the characteristics exhibited by IN718 material in the molten pool of the EA4T steel substrate during laser cladding. The main conclusions are as follows:

1. This study synergistically integrates single-factor experiments, orthogonal designs, grey relational analysis, and signal-to-noise ratio analysis to effectively resolve the conflict between two critical yet competing quality metrics: shape factor and dilution rate.
2. Based on the optimized model, the optimal parameter set was successfully identified (P = 460 W, V_s_ = 7 mm/s, V_f_ = 1.1 r/min), yielding a shape factor of 5.396 and a dilution rate of 29.8%. These values align nearly perfectly with the predefined targets of 5% and 30%, demonstrating the method’s superior efficacy and precision. This ensures the overlay possesses an ideal geometric profile for subsequent overlap and exhibits excellent metallurgical bonding integrity.
3. The systematic determination of an optimal overlap rate of 33.6%, validated by theoretical modeling and experimental flatness measurements, guarantees the formation of uniform, large-area coatings essential for industrial repair applications.
4. By enabling high-quality repair of high-value EA4T components, this approach promotes a circular economy model, reducing material waste, energy consumption, and lifecycle costs associated with full-axle replacement.

## Figures and Tables

**Table 1:** Mass Fraction of Chemical Composition for EA4T Steel (wt%).

C	Si	Mn	P	S	Cr	Ni	Cu	Mo	V	Fe
0.25	0.27	0.68	0.017	0.004	1.09	0.08	0.05	0.23	0.002	allowance

**Table 2:** Mass Fraction of Chemical Composition for Inconel 718 (wt%).

C	Cr	Ni	Co	Mo	Al	Nb	Fe
0.08	20.00	52.00	1.00	3.00	0.5	5.00	allowance

**Table 3:** Model Numbers and Parameters of Major Components.

Component	Model
Robotic Work Platform Number of Motion Axes	KUKA ZH30/60III 6-axis
Robotic Arm	KUKA-KR16-2
Maximum Reach (mm)	1610
Maximum Payload (kg)	16

**Table 4:** Laser Technical Parameters.

Parameter	Value
Operating Voltage (V)	220–380
Laser Beam Wavelength (nm)	1070
Maximum Output Power (W)	500
Geometric Dimensions (mm)	448 × 580 × 132
Wavelength Stability (nm)	±1

**Table 5:** The key Technical Parameters of Powder Feeder.

Technical Parameters	Technical Specifications
Single-Bucket Capacity (L)	1.5
Powder Feed Particle Size (μm)	74–149
Powder Feed Repeatability (%)	<0.3
Carrier Gas Flow Adjustment Range (L/min)	1–20
Carrier Gas Flow Control Method	Digital Flow Control

**Table 6:** Single-Factor Experimental Process Parameter Design Table.

No.	Laser Power P (W)	Scanning Speed V_s_ (mm/s)	Powder Feed Rate V_f_ (r/min)
1	300	5	1
2	340		
3	380		
4	420		
5	460		
6	380	3	1
7		4	
8		5	
9		6	
10		7	
11	380	5	0.8
12			0.9
13			1
14			1.1
15			1.2

**Table 7:** Orthogonal Experimental Design and Measurement Data.

No.	P (W)	V_s_ (mm/s)	V_f_ (r/min)	H (μm)	h (μm)	W (μm)	ξ	η (%)
1	300	3	0.8	382.32	23.41	950.14	2.48	5.77
2	300	4	0.9	251.63	31.21	952.45	3.79	11.03
3	300	5	1.0	215.54	29.26	885.54	4.13	12.00
4	300	6	1.1	146.29	33.16	869.88	5.94	18.48
5	300	7	1.2	159.95	37.06	871.87	5.45	18.81
6	340	3	0.9	450.58	60.46	1039.63	2.31	11.83
7	340	4	1.0	335.48	54.61	1035.67	3.09	14.00
8	340	5	1.1	222.36	68.26	963.51	4.33	23.49
9	340	6	1.2	253.56	78.02	940.15	3.71	23.53
10	340	7	0.8	161.90	64.36	875.74	5.41	28.45
11	380	3	1.0	472.01	79.99	1244.36	2.64	14.49
12	380	4	1.1	325.72	70.22	1191.70	3.66	17.73
13	380	5	1.2	308.17	79.97	1119.58	3.63	20.60
14	380	6	0.8	261.35	91.67	1119.59	4.28	25.97
15	380	7	0.9	220.40	60.46	1086.38	4.93	21.53
16	420	3	1.1	446.65	117.02	1269.84	2.84	20.76
17	420	4	1.2	411.54	130.68	1324.38	3.22	24.10
18	420	5	0.8	314.02	118.98	1267.76	4.04	27.48
19	420	6	0.9	247.70	87.77	1146.94	4.63	26.16
20	420	7	1.0	169.70	91.67	1102.02	6.49	35.07
21	460	3	1.2	462.66	134.71	1508.87	3.26	22.55
22	460	4	0.8	436.90	97.54	1400.62	3.21	18.25
23	460	5	0.9	310.21	89.74	1313.04	4.23	22.44
24	460	6	1.0	237.98	97.54	1233.05	5.18	29.07
25	460	7	1.1	216.53	91.69	1168.49	5.40	29.75

**Table 8:** Analysis of Variance Table for Orthogonal Experiments.

No	P (W)	V_s_ (mm/s)	V_f_ (r/min)	Range Analysis
K_H1_	230.95	442.85	311.30	R_Vs_ > R_P_ > R_Vf_
K_H2_	284.77	352.25	296.10	
K_H3_	317.53	273.86	285.94	
K_H4_	317.92	229.38	271.51	
K_H5_	332.86	185.69	319.17	
R_H_	101.91	257.15	47.66	
K_h1_	30.82	83.12	79.19	R_P_ > R_Vf_ > R_Vs_
K_h2_	95.96	76.85	65.93	
K_h3_	76.46	77.24	70.61	
K_h4_	109.22	77.63	76.07	
K_h5_	102.24	69.05	92.09	
R_h_	78.41	14.07	26.16	
K_W1_	905.97	1202.57	1122.77	R_P_ > R_Vs_ > R_Vf_
K_W2_	970.94	1180.96	1107.69	
K_W3_	1152.32	1109.89	1100.13	
K_W4_	1222.19	1061.92	1092.68	
K_W5_	1324.82	1020.90	1152.97	
R_W_	418.84	181.67	60.29	
K_ξ1_	4.36	2.71	3.88	R_Vs_ > R_Vf_ > R_P_
K_ξ2_	3.77	3.39	3.98	
K_ξ3_	3.83	4.07	4.31	
K_ξ4_	4.25	4.75	4.44	
K_ξ5_	4.26	5.54	3.85	
R_ξ_	0.29	2.83	0.85	
K_η1_	0.13	0.15	0.21	R_P_ > R_Vs_ > R_Vf_
K_η2_	0.20	0.17	0.19	
K_η3_	0.20	0.21	0.21	
K_η4_	0.27	0.25	0.22	
K_η5_	0.24	0.27	0.22	
R_η_	0.14	0.12	0.03	

**Table 9:** Grey Correlation Coefficient Calculation.

No.	Shape Factor GRC	Dilution Rate GRC	Grey Correlation Degree
1#	0.34	0.33	0.34
2#	0.39	0.35	0.37
3#	0.42	0.35	0.38
4#	0.41	0.37	0.39
5#	0.50	0.38	0.44
6#	0.33	0.35	0.34
7#	0.36	0.35	0.36
8#	0.45	0.41	0.43
9#	0.39	0.41	0.40
10#	0.51	0.56	0.53
11#	0.34	0.36	0.35
12#	0.38	0.37	0.38
13#	0.38	0.39	0.38
14#	0.44	0.45	0.45
15#	1.00	0.39	0.70
16#	0.35	0.38	0.37
17#	0.36	0.42	0.39
18#	0.41	0.50	0.45
19#	0.52	0.46	0.49
20#	0.37	0.43	0.40
21#	0.36	0.40	0.38
22#	0.36	0.37	0.37
23#	0.43	0.40	0.42
24#	0.66	0.64	0.65
25#	0.51	1.00	0.76

**Table 10:** Calculation of Height Ratio for Lap Test Specimens.

No	Maximum Height (µm)	Minimum Height (µm)	Aspect Ratio
1	307.25	135.76	0.56
2	328.38	221.30	0.33
3	364.42	214.36	0.41
4	364.50	328.76	0.10
5	371.80	314.63	0.15

## Data Availability

The original contributions presented in the study are included in the article. Further inquiries can be directed to the corresponding author.
